# Colorectal cancer among inflammatory bowel disease patients: risk factors and prevalence compared to the general population

**DOI:** 10.3389/fmed.2023.1225616

**Published:** 2023-08-07

**Authors:** Naim Abu-Freha, Bracha Cohen, Michal Gordon, Sarah Weissmann, Emily H. Kestenbaum, Sergei Vosko, Muhammad Abu-Tailakh, Liza Ben-Shoshan, Daniel L. Cohen, Haim Shirin

**Affiliations:** ^1^The Institute of Gastroenterology and Hepatology, Soroka University Medical Center, Beer-Sheva, Israel; ^2^The Faculty of Health Sciences, Ben-Gurion University of the Negev, Beer-Sheva, Israel; ^3^Soroka Clinical Research Center, Soroka University Medical Center, Beer-Sheva, Israel; ^4^Medical School for International Health, Ben-Gurion University of the Negev, Beer-Sheva, Israel; ^5^The Gonczarowski Family Institute of Gastroenterology and Liver Diseases, Shamir (Assaf Harofeh) Medical Center, The Sackler School of Medicine, Tel Aviv University, Zerifin, Israel; ^6^Recanati School for Community Health Professions, Department of Nursing, Faculty of Health Sciences, Ben-Gurion University of the Negev, Beer-Sheva, Israel; ^7^Soroka University Medical Center, Beer-Sheva, Israel

**Keywords:** colorectal cancer, Crohn’s disease, ulcerative colitis, inflammatory bowel disease, risk factors

## Abstract

**Background:**

Colorectal cancer (CRC) is a feared complication of inflammatory bowel disease (IBD). We aimed to investigate the prevalence and risk factors of CRC among a large cohort of IBD patients.

**Methods:**

Data on IBD patients free of CRC at baseline was extracted using the MDClone platform of the Clalit health maintenance organization in Israel. We investigated the frequency rate of CRC among IBD patients compared to a control group without IBD. Possible risk factors, including comorbidities and IBD-related medications, were investigated in a multivariate analysis.

**Results:**

During a follow-up of 139,448 years among Crohn’s disease (CD) patients and 139,533 years among ulcerative colitis (UC) patients, a frequency rate of CRC was 1.5% (191) among 12,888 CD patients and 2.1% (261) among 12,381 UC patients compared to 1.2% among 57,334 controls. In a multivariate analysis of UC patients, age at diagnosis (OR 1.030, *p* < 0.001), primary sclerosing cholangitis (OR 2.487, *p* = 0.005), diabetes mellitus (OR 2.01, *p* < 0.001), and glucocorticoids treatment (OR 1.465, *p* = 0.008) were found to be predictors of CRC. For CD patients, age at diagnosis (OR 1.035, *p* < 0.001), primary sclerosing cholangitis (OR 2.25, *p* = 0.029), and glucocorticoids treatment (OR 2.07, *p* < 0.001) were found to be predictors for CRC, but not diabetes mellitus.

**Conclusion:**

Despite the continuously decreasing rates of CRC among IBD patients, these are still higher in IBD patients compared to the general population. IBD patients, particularly those with risk factors, require special consideration in follow-up for CRC.

## Introduction

Colorectal cancer (CRC) secondary to long-standing bowel inflammation is one of the feared long-term complications among patients with inflammatory bowel disease (IBD). Studies published five decades ago showed a colitis associated CRC rate of 5 and 40% after 10 and 25 years of disease duration, respectively (1). A meta-analysis published in 2001 by Eaden et al. that included 116 studies with 54,478 ulcerative colitis (UC) patients between 1966 and 1999 found an overall colitis associated CRC prevalence of 3.7% among UC patients. The cumulative risk of colitis associated CRC in this study was 2% after 10 years, 8% at 20 years, and 18% at 30 years (2).

Several disease-related risk factors increased the risk of CRC among IBD patients, including young age at diagnosis, disease duration, severity and extent of disease, the presence of pseudopolyps, primary sclerosing cholangitis (PSC), and a family history of CRC (3–11). Due to the increased risk of colitis associated CRC among IBD patients, it is crucial that patients be followed and undergo surveillance colonoscopies using different methods such as random biopsies, chromoendoscopy and high-resolution endoscopy.

Colonoscopic surveillance should be recommended 8 years after onset of the disease according to the individual risk: annually among high-risk patients (defined as having extensive disease and severe active inflammation, family history of CRC in a first-degree relative ≤50 years, colonic stricture, dysplasia or PSC) (12). Surveillance colonoscopy should be performed every 2–3 years among patients with intermediate risk (defined as having extensive colitis with mild to moderate endoscopic and/or histological inflammation or CRC in first-degree family member >50 years), and every 5 years among low-risk patients (defined as having colitis affecting <50% of the colon, extensive colitis with minimal endoscopic or histological inflammation) (12). In addition, male sex and young age at UC diagnosis are risk factors for CRC among IBD patients (12).

The pathophysiology of colitis associated CRC is different from sporadic CRC. Colitis associated CRC, in which the CRC is related to chronic intestinal inflammation with a long progressive process, begins as indefinite or low-grade dysplasia before progressing to high-grade dysplasia and finally converting to an adenocarcinoma (3). Treatment and follow-up of dysplasia or carcinoma are challenging among IBD patients due to the high-quality of endoscopy required for a thorough examination, the need for total proctocolectomy in cases of multifocal dysplasia, and the evaluation of dysplasia within colonic strictures (3).

Adequate bowel preparation is one of the important factors for high-quality colonoscopies and should be performed under optimal bowel preparation for the disease assessment and surveillance (13).

The rate of colitis associated CRC among IBD patients has continuously decreased. The reduction of colitis associated CRC among IBD patients may be related to two important factors: first, an improved ability to control colonic inflammation in the last three decades due to better anti-inflammatory medication and biological treatment, and second, increasing awareness and improvement of CRC surveillance, including using advanced endoscopic technology. In this study, we aimed to determine the prevalence and risk factors for CRC among IBD patients in a nationwide study.

## Methods

### Patients

This study was a retrospective, population-based, observational study. In this study all adult patients (age 18 years and older) with a confirmed IBD diagnosis were enrolled, who were identified according to the algorithm as described previously (14). The study included data between the years 2000 and 2021, extracted from Clalit, a health maintenance organization in Israel, using a platform powered by MDClone.^1^ Clalit Health Services is one of the largest health maintenance organizations worldwide, with about 4.7 million insured residents, representing over 53% of the Israeli population.

The study was carried out following the principles of the Helsinki Declaration. The study protocol was approved by the Institutional Helsinki Committee, approval number 97–21. Informed consent was waived due to the retrospective design of the study.

### Data collection

Data regarding CD and UC diagnosis were collected. Demographic data such as age, gender, age at diagnosis, BMI, smoking and ethnicity were also collected. Data on chronic diseases (ischemic heart disease, lung disease, diabetes mellitus, hypertension and chronic renal failure) and extraintestinal manifestations (uveitis, scleritis, erythema nodosum, pyoderma ganrenosum), treatments (5-ASA, Azathioprine, mercaptopurine, anti-TNF, methotrexate, glucocorticoids, vedolizumab, ustekinumab) and surgery were collected. Data regarding all-cause mortality was also included. The results were subdivided into CD and UC data and compared between patients with CRC or without CRC diagnosis. CRC diagnoses were identified according to the ICD-9 codes, and only cases diagnosed after the diagnosis of IBD were included.

For comparison of the prevalence of CRC among IBD patients and the general population, a control group from Clalit was used that was matched for age and gender without an IBD diagnosis, no additional exclusions were used for the control group.

### Statistical analysis

Data are presented as mean ± SD for continuous variables and as a percentage (%) of the total for categorical variables. Frequency rates of CRC were calculated among UC and CD patients separately, and the frequency rate of CRC among IBD patients was compared between IBD patients and the general population without IBD-insured patients. Chi-square tests were used to examine univariate relationships between categorical risk factors and the odds of cancer. *T*-tests were used to examine one-variable differences in continuous risk factors that are normally distributed between the group that was ill and those that were not.

Logistic regression models to examine the multivariate relationships between risk factors and the odds of CRC cancer were used. Before introducing the variables into the model, the multicollinearity of the variables was examined using the Variance Inflation Factor (VIF) statistic. The variables found to be significant in the univariate analysis were introduced into the multivariate model one after the other. The order in which the variables entered the model was determined by the size of the univariate Odds Ratio.

All statistical analyses were performed using IBM SPSS version 26 (Chicago, United States). *P*-values less than 0.05 were considered statistically significant.

## Results

### Frequency rate CRC

The frequency rates of CRC among IBD patients compared with the general population are presented in Table 1. The CRC frequency rate was found to be 1.78% among all IBD patients (calculated to be a prevalence of 1780/100,000 IBD cases) compared to 1.23% among the general population (calculated to be a prevalence of 1231/100,000 general population) (*p* < 0.001). A frequency rate of 2.1% among UC patients and 1.5% among CD was found (*p* < 0.001).

**Table 1:** Frequency rate of colorectal cancer among IBD patients versus the general population.

All IBD vs. controls	All IBD *n* = 25,269	Controls *n* = 57,334	*p*-value
All CRCs	452 (1.8)	706 (1.2)	<0.001
UC vs. controls	Ulcerative colitis *n* = 12,381	Controls *n* = 57,334	
CRC among UC	261 (2.1)	706 (1.2)	<0.001
Crohn’s vs. controls	Crohn’s disease *n* = 12,888	Controls *n* = 57,334	
CRC among Crohn’s	191 (1.5)	706 (1.2)	0.022
CRC among UC Vs CD	261 (2.1) 191 (1.5)		<0.001

### Ulcerative colitis patients

UC patients’ characteristics are summarized in Table 2. UC patients diagnosed with CRC were older at the time of IBD diagnosis than UC patients without CRC (57.66 ± 16.3 vs. 44.48 ± 19.3, *p* < 0.001). In addition, higher BMI averages were found among CRC patients, 26.9 ± 4.9 vs. 24.9 ± 5.6 (*p* < 0.001). A significantly higher rate of all-cause mortality among UC CRC patients was found (44.4% vs. 12.8%, *p* < 0.001), and they died at a younger age compared to non-CRC patients (70.17 ± 16.8 vs. 77.98 ± 13.2, *p* < 0.001).

**Table 2:** Characteristics of ulcerative colitis patients with and without colorectal cancer.

Ulcerative colitis	CRC *n* = 261	Non-CRC *n* = 12,120	*p*-value
Gender (male)	132 (50.6)	5,893 (48.6)	0.532
Age, mean ± SD	73.92 ± 15.6	56.6 ± 20.9	<0.001
Age at IBD diagnosis	57.66 ± 16.3	44.48 ± 19.3	<0.001
BMI	26.9 ± 4.9	24.9 ± 5.6	<0.001
Smoking	93 (35.6)	4,192 (34.6)	0.726
Ethnicity – Arab	20 (7.7)	1,441 (11.9)	0.036
Number of hospital Admissions, mean ± SD	9.8 ± 8.3	3.7 ± 6	<0.001
Family history of CRC	25 (9.6)	738 (6.1)	0.019
Death	116 (44.4)	1,553 (12.8)	<0.001
Age at death, mean ± SD	70.17 ± 16.8	77.98 ± 13.2	<0.001
Treatment
5-ASA	215 (82.4)	11,097 (91.6)	<0.001
Azathioprine	24 (9.2)	1,336 (11)	0.350
Mercaptopurine	31 (11.9)	1,250 (10.3)	0.412
Anti-TNF treatment	26 (10)	1,725 (14.2)	0.050
Methotrexate	12 (4.6)	412 (3.4)	0.292
Glucocorticoids treatment	178 (68.2)	6,868 (56.7)	<0.001
Vedolizumab	11 (4.2)	1,119 (9.2)	0.005
Ustekinumab	2 (0.8)	149 (1.2)	0.500

Comorbidities and extraintestinal complications of UC patients with and without CRC are summarized in Supplementary Table S1. Chronic ischemic heart disease (CIHD), diabetes mellitus (DM), obesity, and primary sclerosing cholangitis (PSC) were significantly more common among UC patients with CRC. Table 2 summarizes the medical treatments among UC patients. In general, most of the UC patients were treated with 5-ASA, CRC patients were less frequently treated with 5-ASA than UC patients without CRC (82.4% vs. 91.6%, *p* < 0.001). In contrast, more CRC patients were treated with glucocorticoids (68.2% vs. 56.7%, *p* < 0.001). Only small part of the patients was treated with immunomodulators, about 11% with azathioprine, 11% with mercaptopurine, 14% with anti-TNF, 9% with vedolizumab.

### Univariate and multivariable analysis of CRC among UC patients

**Figure 1:**
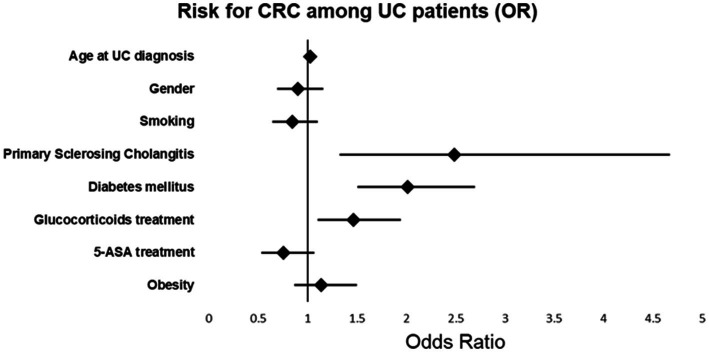
Risk for CRC among UC patients.

Univariate and multivariable cox-regression model analysis of CRC among UC patients is presented in Table 3 and Figure 1. In the multivariate analysis, a significant relationships between the age at diagnosis of UC and the odds of CRC [OR 1.030; *p* < 0.001, 95%CI (1.022, 1.037)], PSC [OR 2.487, *p* = 0.005, 95% CI (1.324, 4.671)], DM [OR 2.016, *p* < 0.001, 95% CI (1.506, 2.699)] and steroid treatment [OR 1.465, *p* < 0.008, 95% CI (1.105, 1.942)] was seen. These were found to be independent risk factors for developing CRC among UC patients.

**Table 3:** Multivariable analysis of CRC risk among UC patients.

	Univariate analysis			Multivariate analysis		
	OR	95% CI	*p*-value	OR	95% CI	*p*-value
Age at UC diagnosis	1.036	1.029–1.043	<0.001	1.030	1.022–1.037	*p* < 0.001
Gender	0.925	0.724–1.183	0.536	0.899	0.696–1.162	0.416
Smoking	1.076	0.833–1.390	0.576	0.846	0.647–1.106	0.204
Primary sclerosing cholangitis	2.471	1.331–4.589	0.004	2.487	1.324–4.671	0.005
Diabetes mellitus	3.502	2.681–4.575	<0.001	2.016	1.506–2.699	<0.001
Glucocorticoids treatment	1.640	1.261–2.133	<0.001	1.465	1.105–1.942	0.008
5-ASA treatment	0.431	0.311–0.596	<0.001	0.754	0.530–1.072	0.116
Obesity	1.629	1.255–2.115	<0.001	1.139	0.867–1.497	0.351
Anti-TNF treatment	0.667	0.443–1.003	0.052	N/A	N/A	N/A
Azathioprine treatment	0.817	0.535–1.249	0.351	N/A	N/A	N/A

### Crohn’s disease patients

The baseline characteristics of CD patients are summarized in Table 4. CD patients with CRC are significantly older than non-CRC patients (68 ± 17 years vs. 50 ± 20 years, *p* < 0.001). In addition, a higher rate of males was found among CRC patients (57.1% vs. 49.7, *p* = 0.042). A non-significant difference regarding smoking was found among the two groups, and a slight yet significantly higher BMI was found (25.6 ± 5.6 vs. 24 ± 5.6, *p* < 0.001). The all-cause mortality rate among CD CRC patients was higher (50.3% vs. 9.4%, *p* < 0.001) and at a younger age (66.23 ± 16.8 years vs. 74.5 ± 15.5 years, *p* < 0.001).

**Table 4:** Characteristics of Crohn’s disease patients with and without colorectal cancer.

Crohn’s disease	CRC *n* = 191	Non-CRC *n* = 12,697	*p*-value
Gender – male	109 (57.1)	6,306 (49.7)	0.042
Age, mean ± SD, years	68 ± 17	50 ± 20	<0.001
Age at IBD diagnosis mean ± SD, years	53.3 ± 17	39.3 ± 19.2	<0.001
Body Mass Index (BMI)	25.6 ± 5.6	24 ± 5.6	<0.001
Smoking	88 (46.1)	5,255 (41.4)	0.192
Ethnicity – Arab	14 (7.3)	1,249 (9.8)	0.247
Number of Admission to hospital, mean ± SD	10.5 ± 8.8	4.74 ± 7	<0.001
Family history of CRC	14 (7.3)	657 (5.2)	0.183
Death	96 (50.3)	1,199 (9.4)	<0.001
Death at age death	66.23 ± 16.8	74.5 ± 15.5	<0.001
Treatment
5-ASA	127 (66.5)	8,245 (64.9)	0.655
Azathioprine	40 (20.9)	2,676 (21.1)	0.964
Mercaptopurine	41 (21.5)	2,446 (19.3)	0.444
Anti-TNF treatment	56 (29.3)	5,124 (40.4)	0.002
Methotrexate	10 (5.2)	838 (6.6)	0.450
Glucocorticoids treatment	150 (78.5)	8,269 (65.1)	<0.001
Vedolizumab	7 (3.7)	1,284 (10.1)	0.003
Usterkinumab	11 (5.8)	1,031 (8.1)	0.235

The comorbidities, extraintestinal manifestations, and complications of CD groups are presented in Supplementary Table S2. Among CD CRC patients, higher rates of CIHD, chronic obstructive lung disease, hypertension, DM, and primary sclerosing cholangitis were found. CD CRC patients had a lower rate of anti-TNF treatment (29.3% vs. 40.4%, *p* < 0.001) but a higher rate of steroid treatment (78.5% vs. 65.1%, *p* < 0.001). The treatment of CD patients is presented in Table 4.

### Univariate and multivariable analysis of CRC among CD patients

**Figure 2:**
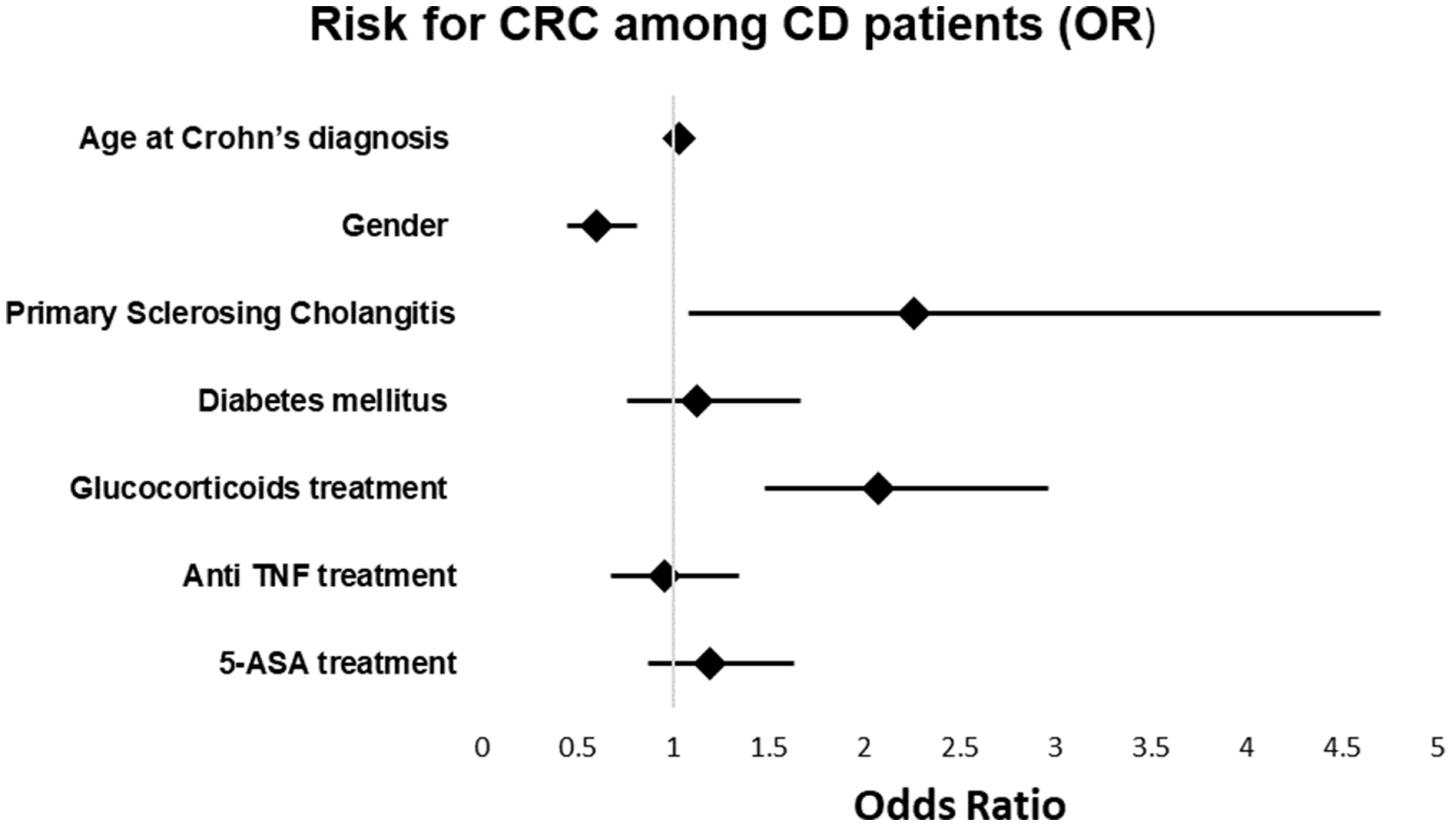
Risk for CRC among CD patients.

In the multivariable model, a relationships between the age at the time of diagnosis of Crohn’s disease and the odds of cancer [OR 1.035, *p* < 0.001; 95%CI (1.027, 1.044)], between primary sclerosing cholangitis and the odds of cancer [OR 2.2, *p* = 0.029; 95%CI (1.086, 4.7)] and between treatment with glucocorticoids and the odds of cancer [OR 2.071, *p* < 0.001; 95%CI (1.479, 2.961)] were found. The relationships between the other variables and the odds of cancer were insignificant. The results of the univariate and multivariable models are summarized in Table 5 and Figure 2.

**Table 5:** Crohn’s disease univariate and multivariate analysis.

Crohn’s disease	Univariate analysis			Multivariate analysis		
	OR	95% CI	*p*-value	OR	95% CI	*p*-value
Age at Crohn’s diagnosis	1.035	1.028–1.042	<0.001	1.035	1.027–1.044	<0.001
Gender	0.753	0.564–1.005	0.054	0.604	0.451–0.810	0.001
Smoking	1.281	0.962–1.706	0.90	N/A	N/A	N/A
Primary sclerosing cholangitis	2.925	1.420–6.023	0.004	2.259	1.086–4.700	0.029
Diabetes mellitus	2.410	1.669–3.482	<0.001	1.129	0.764–1.668	0.544
Obesity	0.866	0.605–1.238	0.429	N/A	N/A	N/A
Glucocorticoids treatment	1.959	1.384–2.773	<0.001	2.071	1.479–2.961	<0.001
Anti TNF treatment	0.613	0.488–0.839	<0.002	0.952	0.675–1.342	0.778
5-ASA treatment	1.071	0.792–1.450	0.655	1.197	0.876–1.634	0.259
Azathioprine treatment	0.992	0.698–1.409	0.964	N/A	N/A	N/A

### Duration of disease and CRC

Finally, the cases were divided into groups based on disease duration of 10 years or less and 11–20 years. For UC, 5,288 patients had ≤10-year disease duration, while 7,089 patients had a disease duration of 11–20 years. Among patients with ≤10 years of disease duration, the frequency rate of CRC was 1.6% (83 cases) compared to 2.5% (178 cases) among patients with a disease duration of 11–20 years, *p* < 0.001. The frequency rate of CRC among CD with ≤10 years duration was 1.3% (75 cases from 5,772 patients), compared to 1.6% (116 cases from 7,116 patients), *p* = 0.122.

## Discussion

We investigated the frequency and risk factors of CRC among a large cohort of patients with IBD. There are several significant findings from this study. First, this study provided confirmation of the known higher frequency rate of CRC among UC and CD patients compared to the general population. Second, CRC patients were found to be older, have higher rates of comorbidities, and have an increased mortality rate compared to IBD patients without CRC. Third, the relationship between age, primary sclerosing cholangitis, steroid treatment, and increased risk of CRC among IBD patients was observed. At the same time, DM was found to be a risk factor for CRC among UC patients but not CD patients.

In this study, a higher frequency rate of CRC among IBD patients was found in comparison to the general population. This can be attributed to the pathogenesis of the disease and its relationship to chronic inflammation of the colon. Our findings agree with a previous study from Canada, in which an increase in incidence rates of CRC in UC patients was found (with an odds ratio of 2.75) (15). However, a decline in CRC incidence and mortality among IBD patients was observed in other studies (16). The decline in CRC incidence could be due to the general improvement of screening among patients at screening age groups or due to the major advances in the treatment and management of inflammation of IBD patients in the last three decades. This may explain why the incidence rate of CRC in UC patients was 2.1% in our study, which is less than in the study from Canada. Despite improvements, CRC rates are still higher in IBD patients compared to the general population, and IBD patients require special consideration in follow-up and surveillance.

In the present study, the age of CRC patients was significantly higher than those without CRC, the age of IBD diagnosis was also older in CRC patients, and there was a significant relationship between age and development of CRC among both UC and CD. The aging process and increased disease duration could impact the pathogenesis of CRC development in terms of additive risk for CRC and exacerbation of chronic inflammation.

In this study, we also focused on comorbidities and risk factors for developing CRC. IBD patients who developed CRC had a higher rate of comorbidities such as metabolic syndrome, DM, and obesity. This makes sense as these comorbidities are also known to increase the risk for CRC (17–20).

Concerning all-cause mortality, CRC increased the mortality rate significantly among IBD patients, reaching 44–50% in CRC IBD patients compared to 12 and 9% among IBD patients without CRC. Our results agree with previous studies that showed an increased all-cause mortality rate (21, 22). Infections, cancer, and emergent surgery complications seem to be the most common causes of mortality in IBD patients (22–24). High rates of metabolic syndrome were found, which may be a contributing factor to the high all-cause mortality rates. It is important to note that CRC patients in our study have higher rates of comorbidities compared to previous studies (25). Another previous report showed that increased mortality rates are correlated with CRC among IBD patients (26).

With regard to specific comorbidities and treatments, primary sclerosing cholangitis and glucocorticoid treatment increased the risk for CRC among UC and CD patients. Primary sclerosing cholangitis is known to be a risk factor for CRC among IBD patients (27). In our large cohort, the multivariable analysis showed a relationship between glucocorticoid steroid treatment, DM, and CRC. Glucocorticoid treatment is a common treatment among IBD patients, particularly during disease exacerbations. The association between glucocorticoids and CRC, in general, is controversial: one previous study found no substantial association between the two (28); however, other studies showed an increased risk of 1.4-fold of CRC among patients treated with glucocorticoids. The mechanism of glucocorticoids on CRC could be related to the suppression of the host’s immune system and impact on colorectal cancer cells. The impact of glucocorticoids signaling on intestinal tumorigenesis remains controversial (29, 30); however, one previous study demonstrated that intestinal epithelial GR signaling repressed acute colitis but promoted chronic inflammation-associated colorectal cancer (31). Alternatively, using steroids may be a surrogate representing either a more severe or poorly controlled disease. Regardless, glucocorticoids are known to have several short and long-term side effects and should be used as little as possible. Additional studies are needed in order to investigate the relationship between glucocorticoids and future CRC development.

A relationship between DM and CRC diagnosis among UC patients was demonstrated but not among CD patients. DM is known to be a risk factor for CRC. Previous studies, systematic reviews, and meta-analyses have reported an increased risk of CRC among patients with DM compared to non-diabetics (32, 33). A recently published meta-analysis showed an increased risk for IBD-related hospitalizations (OR = 2.52, *p* < 0.00001) and infections (sepsis OR = 1.56, pneumonia OR = 1.72 and urinary tract infections OR = 1.93) among IBD patients with DM, but no significant increased risk for IBD related complication (OR 1.12, *p* = 0.77), IBD-related surgery (OR = 1.2, *p* = 0.26) or mortality (OR = 1.52, *p* = 0.37) (34). Further investigations are needed to elucidate the risk of DM on CRC development among IBD patients. While DM increased the risk of CRC among general population, we found DM could be a risk factor CRC among UC but not CD patients. It is possible that DM may affect the course of the disease (34), and IBD patients’ exposure to glucocorticoids in combination with glucocorticoids induced DM could be the risk factor for CRC.

In our study, univariate analysis showed that anti-TNF treatment decreases the risk for CRC among IBD patients. This trend was significant for CD patients but not significant for UC patients. These findings are in agreement with previous studies, which showed that patients treated with anti-TNF agents are less likely to develop CRC (35, 36). The protective effect of anti-TNF treatment could be related to better control of chronic inflammation with clinical remission and mucosal healing.

Our results showed that UC patients of Arab origin had a lower frequency rate of CRC. Other studies also showed different prevalence of colonic dysplasia among Hispanics compared to non-Hispanic white’ counterparts, despite similar risk factors (37). Racial disparities were reported in the overall incidence of CRC and patients with IBD (38). Differences in CRC prevalence among IBD patients in relation to ethnicity could be explained by different exposures to risk factors such smoking, alcohol consumption, dietary habits and to the characteristics, course, and treatment of IBD (37, 38). In a recently published study, Arab UC patients from a large cohort in Israel were younger in age, younger at the time of UC diagnosis and had a lower rate of smoking and a higher rate of anti-TNF treatment than their Jewish counterparts (39). All of these factors could explain the lower CRC rate found among Arab UC patients found in our study.

In summary, our study focused on risk factors of CRC development among IBD patients in terms of comorbidities and treatments. In addition to the known risk factors of age and PSC, a significant relationships between glucocorticoid treatment and CRC diagnosis and diabetes and CRC development among UC patients were demonstrated.

The strength of our study lies in the large number of patients included and length of follow-up time (two decades). However, despite these strengths, there are several limitations. First, this study used a retrospective design. Second, there was no data regarding the IBD severity, CRC stage, CRC localization, or CRC treatment. Finally, no data regarding the specific cause of death was available.

## Conclusion

IBD patients have a higher risk for CRC than the general population, with age, PSC, glucocorticoid use as the main risk factors, and diabetes mellitus among UC patients. Additional studies are needed to investigate further the association between glucocorticoid treatment and diabetes mellitus and CRC risk. IBD patients, particularly those with risk factors, require special consideration in follow-up for CRC.

## Data availability statement

The data analyzed in this study is subject to the following licenses/restrictions: no additional data are available. Requests to access these datasets should be directed to abufreha@yahoo.de.

## Ethics statement

The studies involving human participants were reviewed and approved by the Soroka Helsinki Committee. Written informed consent for participation was not required for this study in accordance with the national legislation and the institutional requirements.

## Author contributions

NA-F: conceptualization, methodology, supervision, writing original draft, and project administration. BC and MG: software, resources, and writing review and editing. SW: software, resources, data curation, formal analysis, and writing—review and editing. EK: resources, data curation, formal analysis, and writing—review and editing. SV: methodology, validation, and writing—review and editing. MA-T: validation, data curation, formal analysis, and writing—review and editing. LB-S: data curation, resources, and writing—review and editing. DC: methodology, resources, and writing—review and editing. HS: methodology, validation, supervision, and writing—review and editing. All authors contributed to the article and approved the submitted version.

## Conflict of interest

The authors declare that the research was conducted in the absence of any commercial or financial relationships that could be construed as a potential conflict of interest.

## Publisher’s note

All claims expressed in this article are solely those of the authors and do not necessarily represent those of their affiliated organizations, or those of the publisher, the editors and the reviewers. Any product that may be evaluated in this article, or claim that may be made by its manufacturer, is not guaranteed or endorsed by the publisher.

## Footnotes

^1^https://www.mdclone.com
