# Supplementary material for: Colorectal cancer among inflammatory bowel disease patients: risk factors and prevalence compared to the general population
Source: Front Med (Lausanne). 2023 Aug 7;10:1225616. doi: 10.3389/fmed.2023.1225616 (PMC10443703; doi:10.3389/fmed.2023.1225616)
Supplement: Supplementary file 1 [file Table_1.DOCX]

Table S1: Comorbidities, extraintestinal manifestation and complications of ulcerative colitis patients

| **Ulcerative Colitis** | CRC  n=261 | Non-CRC  n=12120 | p-value |
| --- | --- | --- | --- |
| CIHD | 87 (33.3) | 2135 (17.6) | <0.001 |
| CHF | 30 (11.5) | 746 (6.2) | <0.001 |
| COPD | 33 (12.6) | 1100 (9.1) | 0.048 |
| Asthma | 51 (19.5) | 1741 (14.4) | 0.019 |
| Chronic renal failure | 65 (24.9) | 1293 (10.7) | <0.001 |
| Hypertension | 159 (60.9) | 4025 (33.2) | <0.001 |
| Diabetes mellitus | 82 (31.4) | 1402 (11.6) | <0.001 |
| Dyslipidemia | 158 (60.5) | 4961 (40.9) | <0.001 |
| NAFLD | 31 (11.9) | 955 (7.9) | 0.018 |
| Obesity | 87 (33.3) | 2846 (23.5) | <0.001 |
| CVA | 16 (6.1) | 290 (2.4) | <0.001 |
| Dementia | 23 (8.8) | 454 (3.7) | <0.001 |
| Liver cirrhosis | 9 (3.4) | 141 (1.2) | 0.001 |
| Vitamin B12 deficiency | 9 (3.4) | 170 (1.4) | 0.006 |
| Folic acid deficiency | 79 (30.3) | 2822 (23.3) | 0.008 |
| Iron deficiency anemia | 146 (55.9) | 4696 (38.7) | <0.001 |
| Vitamin D deficiency | 62 (23.8) | 2704 (22.3) | 0.579 |
| Primary sclerosing cholangitis | 11 (4.2) | 212 (1.7) | 0.003 |
| Arthritis | 84 (32.2) | 2271 (18.7) | <0.001 |
| Osteoporosis | 45 (17.2) | 892 (7.4) | <0.001 |
| Uveitis | 30 (11.5) | 789 (6.5) | 0.001 |
| Scleritis | 3 (1.1) | 132 (1.1) | 0.926 |
| Erythema Nodosum | 2 (0.8) | 155 (1.3) | 0.464 |
| Pyoderma gangrenosum | 1 (0.4) | 35 (0.3) | 0.779 |
| Pancreatitis | 9 (3.4) | 293 (2.4) | 0.285 |
| Peri-anal abscess | 10 (3.8) | 354 (2.9) | 0.389 |
| Anal fissure | 7 (2.7) | 258 (2.1) | 0.5411 |
| Pulmonary embolism | 12 (4.6) | 156 (1.3) | <0.001 |
| DVT | 13 (5) | 160 (1.3) | <0.001 |

CIHD=Chronic Ischemic Heart disease, CHF=Congestive Heart disease, COPD=chronic obstructive pulmonary disease, NAFLD=Non alcoholic fatty liver disease, CVA=Cerebrovascular accident, DVT=Deep Venous Thrombosis
