# Supplementary material for: Colorectal cancer among inflammatory bowel disease patients: risk factors and prevalence compared to the general population
Source: Front Med (Lausanne). 2023 Aug 7;10:1225616. doi: 10.3389/fmed.2023.1225616 (PMC10443703; doi:10.3389/fmed.2023.1225616)
Supplement: Supplementary file 2 [file Table_2.DOCX]

Table S2: Comorbidities, extraintestinal manifestation and complications of Crohn’s disease patients

| **Crohn’s disease** | CRC  n=191 | Non-CRC  n=12697 | p-value |
| --- | --- | --- | --- |
| CIHD | 46 (24.1) | 1739 (13.7) | <0.001 |
| CHF | 22 (11.5) | 615 (4.8) | <0.001 |
| COPD | 33 (17.3) | 1089 (8.6) | <0.001 |
| Asthma | 26 (13.6) | 2024 (15.9) | 0.383 |
| Chronic renal failure | 51 (26.7) | 1220 (9.6) | <0.001 |
| Hypertension | 92 (48.2) | 3440 (27.1) | <0.001 |
| Diabetes mellitus | 36 (18.8) | 1116 (8.8) | <0.001 |
| Dyslipidemia | 93 (48.7) | 3970 (31.3) | <0.001 |
| NAFLD | 12 (6.3) | 1057 (8.3) | 0.310 |
| Obesity | 38 (19.9) | 2831 (22.3) | 0.428 |
| CVA | 8 (4.2) | 260 (2) | 0.040 |
| Dementia | 15 (7.9) | 347 (2.7) | <0.001 |
| Liver cirrhosis | 6 (3.1) | 139 (1.1) | 0.008 |
| Vitamin B12 deficiency | 11 (5.8) | 252 (2) | <0.001 |
| Folic acid deficiency | 63 (33) | 3878 (30.5) | 0.467 |
| Iron deficiency anemia | 119 (62.3) | 5913 (46.6) | <0.001 |
| Vitamin D deficiency | 53 (27.7) | 2984 (23.5) | 0.170 |
| Primary sclerosing cholangitis | 8 (4.2) | 187 (1.5) | 0.002 |
| Arthritis | 42 (22) | 1993 (15.7) | 0.018 |
| Osteoporosis | 22 (11.5) | 953 (7.5) | 0.037 |
| Uveitis | 21 (11) | 653 (5.1) | <0.001 |
| Scleritis | 5 (2.6) | 202 (1.6) | 0.262 |
| Erythema Nodosum | 5 (2.6) | 287 (2.3) | 0.742 |
| Pyoderma gangersum | 2 (1) | 23 (0.2) | 0.007 |
| Pancreatitis | 5 (2.6) | 412 (3.2) | 0.627 |
| Peri-anal abscess | 14 (7.3) | 959 (7.6) | 0.908 |
| Anal fissure | 12 (6.3) | 638 (5) | 0.430 |
| Pulmonary embolism | 4 (2.1) | 203 (1.6) | 0.589 |
| DVT | 10 (5.2) | 207 (1.6) | <0.0012 |
| **Surgery** |  |  |  |
| Right Hemicolectomy | 20 (10.5) | 331 (2.6) | <0.001 |
| Left hemicolectomy | 2 (1) | 14 (0.1) | <0.001 |
| Total colectomy | 4 (2.1) | 29 (0.2) | <0.001 |
| Subtotal colectomy | 1 (0.5) | 1 | <0.0011 |
| Small bowel resection | 12 (6.3) | 319 (2.5) | 0.001 |
| Anterior resection | 8 (4.2) | 9 (0.1) | <0.001 |
| Ileostomy | 2 (1) | 14 (0.1) | <0.001 |
| Colostomy | 2 (1) | 19 (0.1) | 0.002 |
| Abdominal abscess | 5 (2.6) | 339 (2.7) | 0.965 |

CIHD=Chronic Ischemic Heart disease, CHF=Congestive Heart disease, COPD=chronic obstructive pulmonary disease, NAFLD=Non alcoholic fatty liver disease, CVA=Cerebrovascular accident, DVT=Deep Venous Thrombosis
